# Supplementary material for: Prognostic Value of Immune Environment Analysis in Small Bowel Adenocarcinomas with Verified Mutational Landscape and Predisposing Conditions
Source: Cancers (Basel). 2020 Jul 23;12(8):2018. doi: 10.3390/cancers12082018 (PMC7463640; doi:10.3390/cancers12082018)
Supplement: Supplementary file 1 [file cancers-12-02018-s001.pdf]

Supplementary Materials:

Prognostic Value of Immune Environment Analysis in Small Bowel Adenocarcinomas with Verified Mutational Landscape and Predisposing Conditions

Table S1a. Correlations between immune parameters and different mutation types and detected mutational signatures in MSS tumours.

		ICS	PD-1	PD-L1^{IC}	Immunoprofile
Synonymous	r_s	-0.114	-0.118	-0.044	-0.093
	P	0.317	0.302	0.701	0.418
Nonsynonymous	r_s	-0.065	-0.069	0.033	-0.008
	P	0.569	0.548	0.776	0.945
Missense	r_s	-0.066	-0.063	0.044	-0.003
	P	0.562	0.581	0.701	0.978
Splicesite	r_s	-0.035	-0.183	-0.042	-0.074
	P	0.759	0.107	0.712	0.519
Nonsense	r_s	-0.058	-0.060	-0.067	-0.041
	P	0.612	0.602	0.555	0.724
FrameShift	r_s	0.033	-0.042	0.039	0.048
	P	0.772	0.713	0.731	0.675
Inframe	r_s	-0.012	0.150	-0.062	0.028
	P	0.919	0.188	0.588	0.805
Signature 1A	r_s	-0.199	-0.146	-0.096	-0.184
	P	0.078	0.200	0.399	0.107
Signature 17	r_s	0.146	0.073	0.318	0.269
	P	0.199	0.523	0.004	0.017
Signature U2	r_s	-0.006	0.115	0.099	0.081
	P	0.958	0.314	0.385	0.479

Abbreviations: ICS, immune cell score; PD-1, programmed cell death 1; PD-L1^{IC}, programmed death ligand 1 positive immune cells; r_s, Spearman correlation coefficient. The mutation types include all somatic changes in the coding regions detected by exome sequencing in MSS tumours.

Supplementary Table S1b. Correlations between immune parameters and different mutation types in MSI tumours.

		ICS	PD-1	PD-L1^{IC}	Immunoprofile
Synonymous	r_s	-0.471	-0.405	-0.166	-0.381
	P	0.089	0.151	0.570	0.179
Nonsynonymous	r_s	-0.392	-0.405	-0.166	-0.323
	P	0.165	0.151	0.570	0.260
Missense	r_s	-0.392	-0.405	-0.203	-0.352
	P	0.165	0.151	0.486	0.217
Splicesite	r_s	-0.216	-0.279	-0.056	-0.136
	P	0.458	0.334	0.850	0.642
Nonsense	r_s	-0.353	-0.253	-0.018	-0.215
	P	0.216	0.382	0.950	0.461
FrameShift	r_s	-0.275	-0.101	-0.018	-0.193
	P	0.342	0.730	0.950	0.509
Inframe	r_s	-0.118	0.051	0.167	0.031
	P	0.687	0.863	0.568	0.915

Abbreviations: ICS, immune cell score; PD-1, programmed cell death 1; PD-L1^{IC}, programmed death ligand 1 positive immune cells; r_s, Spearman correlation coefficient. The mutation types include all somatic changes in the coding regions detected by exome sequencing in MSI tumours.

Table S2. Survival according to clinicopathological variables.

	Disease-Specific Survival				Overall Survival			
	**N**	**5-Year Survival**	**10-Year Survival**	***p*-Value**	**N**	**5-year survival**	**10-Year Survival**	***p*-Value**
Age:								
<60 years	37	43%	37%	0.611	37	43%	34%	0.637
≥60 years	50	49%	42%		50	40%	30%	
Sex:								
female	40	48%	40%	0.827	40	43%	36%	0.533
male	47	45%	40%		47	40%	29%	
TNM stage:								
I	4	75%	75%	<0.001	4	75%	25%	<0.001
II	18	67%	61%		18	67%	55%	
III	24	70%	60%		24	67%	57%	
IV	31	7%	7%		31	7%	7%	
Tumour grade								
1	16	41%	14%	0.397	16	31%	10%	0.530
2	53	52%	47%		53	47%	37%	
3	15	40%	40%		15	40%	40%	
Immune cell score								
0	26	29%	24%	0.009	26	23%	15%	0.002
1	12	37%	24%		12	33%	22%	
2	15	24%	24%		15	20%	20%	
3	10	69%	41%		10	60%	36%	
4	24	71%	71%		24	71%	62%	
Immune cell score								
low	53	30%	24%	<0.001	53	25%	18%	<0.001
high	34	70%	63%		34	68%	55%	
PD-1^{high}								
no	60	31%	24%	<0.001	60	27%	18%	<0.001
yes	27	80%	76%		27	74%	62%	
PD-L1^{IChigh}								
no	64	33%	26%	<0.001	64	28%	20%	<0.001
yes	22	81%	76%		22	77%	63%	
PD-L1^{TChigh}								
no	79	49%	42%	0.060	79	43%	33%	0.137
yes	7	14%	14%		7	14%	14%	
Immunoprofile:								
0	43	26%	18%	<0.001	43	21%	12%	<0.001
1	17	36%	27%		17	29%	22%	
2	12	73%	73%		12	67%	67%	
3	14	93%	85%		14	93%	71%	
MMR status:								
MSS	73	39%	31%	0.003	73	34%	23%	0.001
MSI	14	85%	85%		14	79%	79%	
TMB								
low	69	39%	32%	0.009	69	33%	23%	0.003
high	18	77%	70%		18	72%	66%	
Tumour location:								
duodenal	17	50%	50%	0.034	17	47%	29%	0.010
jejunum	47	52%	48%		47	51%	44%	
ileum	14	34%	11%		14	21%	7%	
Coeliac disease:								
no	78	42%	35%	0.045	78	37%	27%	0.014
yes	9	78%	78%		9	78%	78%	

Table S3. Multivariable analysis with Immune cell score, PD-1 and PD-L1^{IC}, and without MMR status.

	**Univariable Analysis**	**Disease-Specific Survival (*n* = 68)**		**Overall Survival (*n* = 68)**	
	***p*-Value**	**HR (95% CI)**	***p*-Value**	**HR (95% CI)**	***p*-Value**
Age					
<60 years	DSS: 0.614	1	0.247	1	0.170
≥60 years	OS: 0.640	1.63 (0.71–3.74)		1.71 (0.80–3.68)	
Sex					
Female	DSS: 0.829	1	0.217	1	0.348
Male	OS: 0.536	0.63 (0.30–1.31)		0.71 (0.35–1.45)	
TNM stage					
I		1		1	
II	DSS: <0.001	2.15 (0.20–23.39)	<0.001	0.76 (0.14–4.04)	<0.001
III	OS: <0.001	3.07 (0.31–30.86)		0.97 (0.20–4.78)	
IV		16.35 (1.39–191.99)		6.58 (1.11–38.92)	
Immune cell score					
low	DSS: 0.001	2.01 (0.79–5.10)	0.142	2.43 (0.98–6.06)	0.056
high	OS: <0.001	1		1	
PD-1					
low	DSS: <0.001	1.76 (0.44–7.09)	0.426	0.99 (0.29–3.40)	0.988
high	OS: <0.001	1		1	
PD-L1^{IC}					
low	DSS: <0.001	5.87 (1.55–22.28)	0.009	4.94 (1.55–15.69)	0.007
high	OS: <0.001	1		1	
Tumour location					
Duodenum	DSS: 0.043	1	<0.001	1	0.001
Jejunum	OS: 0.015	0.79 (0.23–2.65)		0.73 (0.24–2.22)	
Ileum		6.96 (1.40–34.61)		5.03 (1.13–22.37)	

Abbreviations: HR, hazard ratio; CI, confidence interval; DSS, disease-specific survival; OS, Overall survival; PD-1, programmed cell death protein 1; PD-L1, programmed death ligand 1; IC, immune cell. Analyses were performed with the following reference categories: <60 years, Female gender, TNM Stage I, high Immune cell score, high PD-1, high PD-L1^{IC}, and duodenal tumour location. For analyses there were 68 patients available. Eleven patients were excluded because of unknown TNM stage, one patient had insufficient samples for Immune cell score and PD-1, one patient had insufficient samples for PD-L1^{IC}, nine patients had unknown tumour location, three patients had insufficient survival data, and two patients were excluded because of postoperative death.

Table S4. Multivariable analysis with Immunoprofile and without MMR status.

	Univariable analysis	Disease-specific survival (n = 68)		Overall survival (n = 68)	
	p-Value	HR (95% CI)	*p*-Value	HR (95% CI)	*p*-Value
Age					
<60	DSS: 0.614	1	0.075	1	0.061
≥60	OS: 0.640	2.12 (0.93–4.85)		2.10 (0.97–4.54)	
Sex					
Female	DSS: 0.829	1	0.042	1	0.068
Male	OS: 0.536	0.47 (0.23–0.97)		0.52 (0.26–1.05)	
TNM stage					
I		1		1	
II	DSS: <0.001	2.61 (0.25–26.94)	<0.001	1.02 (0.20–5.25)	<0.001
III	OS: <0.001	5.43 (0.57–51.44)		1.89 (0.41–8.65)	
IV		27.18 (2.38–310.95)		10.88 (1.85–63.90)	
Immunoprofile					
Low	DSS: <0.001	11.96 (3.39–42.15)	<0.001	7.54 (2.75–20.67)	<0.001
High	OS: <0.001	1		1	
Tumour location					
Duodenum		1		1	
Jejunum	DSS: 0.043<br>OS: 0.015	0.60 (0.18–2.00)	0.002	0.53 (0.17–1.63)	0.004
Ileum		3.28 (0.76–14.14)		2.35 (0.59–9.38)	

Abbreviations: HR, hazard ratio; CI, confidence interval; DSS, disease-specific survival; OS, Overall survival. Analyses were performed with the following reference categories: <60 years, female gender, TNM Stage I, high Immunoprofile, and duodenal tumour location. For analyses there were 68 patients available. Eleven patients were excluded because of unknown TNM stage, two patients were excluded because of insufficient samples for Immunoprofile, nine patients had unknown tumour location, three patients had insufficient survival data, and two patients were excluded because of postoperative death.

Table S5. Multivariable analysis with coeliac and Crohn's disease.

	Univariable Analysis	Disease-Specific Survival (*n* = 68)		Overall Survival (*n* = 68)	
	p-Value	HR (95% CI)	*p*-Value	HR (95% CI)	*p*-Value
Age					
<60 years	DSS: 0.614	1	0.097	1	0.044
≥60 years	OS: 0.640	2.17 (0.87–5.40)		2.41 (1.02–5.69)	
Sex					
Female	DSS: 0.829	1	0.250	1	0.411
Male	OS: 0.536	0.66 (0.32–1.35)		0.75 (0.38–1.49)	
TNM stage					
I		1		1	
II	DSS: <0.001	3.66 (0.31–43.72)	0.001	1.03 (0.17–6.20)	<0.001
III	OS: <0.001	6.61 (0.54–80.41)		1.57 (0.25–9.80)	
IV		29.57 (2.21–395.27)		10.27 (1.50–70.46)	
MMR status					
MSS	DSS: 0.010	2.90 (0.26–33.13)	0.391	8.45 (0.81–88.24)	0.075
MSI	OS: 0.004	1		1	
Immune cell score					
low	DSS: 0.001	1.76 (0.69–4.53)	0.239	2.14 (0.87–5.27)	0.097
high	OS: <0.001	1		1	
PD-1					
low	DSS: <0.001	2.04 (0.40–10.37)	0.390	0.81 (0.20–3.28)	0.768
high	OS: <0.001	1		1	
PD-L1^{IC}					
low	DSS: <0.001	4.83 (1.21–19.32)	0.026	3.51 (1.12–11.04)	0.032
high	OS: <0.001	1		1	
Tumour location					
Duodenum	DSS: 0.043	1	<0.001	1	0.001
Jejunum	OS: 0.015	0.57 (0.15–2.27)		0.71 (0.20–2.54)	
Ileum		5.03 (0.95–26.78)		5.20 (1.07–25.30)	
Coeliac disease					
No	DSS: 0.020	6.12 (0.62–60.34)	0.121	5.59 (0.63–49.89)	0.124
Yes	OS: 0.003	1		1	
Crohn's disease					
No	DSS: 0.175	1.21 (0.31–4.66)	0.782	0.90 (0.24–3.42)	0.882
Yes	OS: 0.300	1		1	

Abbreviations: HR, hazard ratio; CI, confidence interval; DSS, disease-specific survival; OS, Overall survival; MMR, Mismatch repair; MSS, microsatellite stable; MSI, microsatellite instable; PD-1, programmed cell death protein 1; PD-L1, programmed death ligand 1; IC, immune cell. Analyses were performed with the following reference categories: <60 years, Female gender, TNM Stage I, MSI status, high Immune cell score, high PD-1, high PD-L1^{IC}, duodenal tumour location, coeliac disease, and Crohn's disease. For analyses there were 68 patients available. Eleven patients were excluded because of unknown TNM stage, one patient had insufficient samples for Immune cell score and PD-1, one patient had insufficient samples for PD-L1^{IC}, nine patients had unknown tumour location, three patients had insufficient survival data, and two patients were excluded because of postoperative death.
